# Physicochemical Stability and Compatibility Testing of Voriconazole in All-in-One Parenteral Nutrition Admixtures

**DOI:** 10.3390/pharmaceutics13091447

**Published:** 2021-09-10

**Authors:** Emilie Reber, Peter Neyer, Katja A. Schönenberger, Christoph Saxer, Luca Bernasconi, Zeno Stanga, Andreas Huber, Angelika Hammerer-Lercher, Stefan Mühlebach

**Affiliations:** 1 Department of Diabetes, Endocrinology, Nutritional Medicine and Metabolism, Inselspital, Bern University Hospital, University of Bern, 3010 Bern, Switzerland; katja.schoenenberger@extern.insel.ch (K.A.S.); zeno.stanga@insel.ch (Z.S.); 2 Institute of Laboratory Medicine, Kantonsspital Aarau, 5001 Aarau, Switzerland; peter.neyer@ksa.ch (P.N.); christoph.saxer@ksa.ch (C.S.); luca.bernasconi@ksa.ch (L.B.); andreas.huber@ufl.li (A.H.); angelika.hammerer@ksa.ch (A.H.-L.); 3 Division of Clinical Pharmacy and Epidemiology and Hospital Pharmacy, University of Basel, 4031 Basel, Switzerland; stefan.muehlebach@unibas.ch

**Keywords:** parenteral nutrition, voriconazole stability and compatibility, lipid droplet microscopy, LC–MS/MS

## Abstract

(1) Drug compatibility with all-in-one (AiO) parenteral nutrition (PN) admixtures is a very important pharmaceutical quality issue to be answered based on appropriate laboratory testing. We assessed voriconazole (V), a poorly water-soluble (logP ≈ 1) single-daily dosed antifungal drug monitored in patients and thus candidate for AiO PN admixing for convenient and safe patient care. We evaluated V compatibility and stability in AiO PN admixtures through adapted therapeutic drug monitoring method (drug stability) and visual microscopic emulsion stability by lipid droplets analysis improved by an automated microscopic digital assessment. (2) V was added in concentrations of 0.05/0.25/0.5 mg/mL (143.1/715.7/1431.5 µM), correlating to daily therapeutic dosing, to three commercially available industrial AiO PN admixtures. Three aliquots were stored in the refrigerator (4 °C), at room temperature (24 °C) and under stress conditions in a water bath (37 °C). Samples taken at 0/24/48/72/168 h after admixing were subjected to a stability-indicating one-week analysis. Assessment included visual examination, lipid droplet measurement according to an established and validated method (bright-field microscopy using oil immersion), pH measurement (glass electrode) and V identification/quantification (LC–MS/MS). (3) After one week, all samples at 37 °C showed slight yellow discoloration. The pH values remained stable. All samples met specifications for lipid droplets according to size (upper size ≤8 µm, mean size <4.5 ± 2 µm) and number (*n* ≤ 9 lipid droplets >5 µm). V concentrations were within an acceptable range, calculated for every timepoint as percent of the theoretical concentration spiked into the AiO PN. The median recovery was 98.2% (min–max, 90–112%). (4) At therapeutic doses, commercial V formulations were compatible and stable within specifications over one week in commonly used volumes of commercial AiO PN admixtures at 4–37 °C.

## 1. Introduction

Parenteral nutrition (PN) is indicated whenever the function of the gastrointestinal tract is impaired for adequate enteral nutrition [1]. PN is a life-saving therapy indicated in many clinical settings, e.g., for neonates and critically ill patients, and can even be administered at home for the long term. Although not recommended, mainly due to potential stability and sterility issues, coadministration of drugs and PN may be requested to simplify treatment since intravenous access is often limited. All-in-one (AiO) PN admixtures are complex, mostly oil-in-water emulsions with limited stability. Such admixtures contain up to 50 individual components, which can easily interact with each other or with the container (multilayer bag material consisting of ethyl–vinyl acetate of different vinyl- and polyamide copolymers) or the (semisynthetic) butyl rubber stopper, potentially allowing ad- and absorption or gas permeation [2,3]. Individualising or preparing the ready-to-use commercial AiO PN by adding vitamins, trace elements, electrolytes (especially di- and trivalent cations critical for stability issues, e.g., Mg^2+^, Ca^2+^, Fe^3+^) or medication further increases the potential risk of physicochemical instability including emulsion destabilization, hampering the nutritional and therapeutic effect and safety [4,5]. Light exposure and storage temperature may, among others, potentially further affect the emulsion stability, the risk of precipitation, or chemical degradation [6]. Lipid droplet enlargement, coalescence and resulting emulsion creaming or even breaking are eventually highly critical instability problems of PN admixtures affecting the overall quality characteristics and safety of such treatments. Chemical reactions may be accompanied by (visible) colour (polymerisation, (per-)oxidation) and/or measurable pH changes (triglyceride hydrolysis), potentially also causing precipitation due to altered solubility parameters. These instabilities and incompatibilities can lead to major clinical, potentially lethal complications, e.g., venous catheter occlusion or blood vessel obstruction (emboli) due to precipitates or enlarged lipid droplets (>5 μm) [7,8,9,10,11]. The importance to quantify the enlarged fat droplets population (>2–5 μm in diameter) was also stressed by the work of Driscoll and colleagues when introducing pharmacopoeial stability parameters [7,11]. Therefore, from a quality standpoint, a single particle measure is necessary to assess the amount of fat infused with enlarged fat droplets, e.g., the volume-weighted percent of fat droplets greater than five micrometres (PFAT5) must not exceed 0.05% of the total fat, as stated in the United States Pharmacopeia [11].

In cases where PN regimens are individualised and, where inevitable, comedication is added directly or coadministered via a Y-site, PN quality, efficacy and safety remain mostly unclear. Such AiO PN admixtures represent new formulations; therefore, no liability of the original manufacturer on quality, efficacy and safety applies (changed formulation/composition). Documentation of stability and compatibility, at least on short-term usage, is mandatory to guarantee quality, safety, tolerance and, finally, efficacy [4]. Administering such regimens without documented compatibility and stability has, thus, to be considered an avoidable medication error. Given the high number of components and the complexity of AiO PN emulsions, evidence level on the correlation between physicochemical drug characteristics including drug formulation ingredients (e.g., solubilisers) and PN compatibility and stability must increase. Ultimately, one should aim for an initial stability algorithm to assess potential compatibility and stability of AiO drug admixtures, requesting general evaluation of given physicochemical data of related drug classes such as hydrophilic or lipophilic drugs and their physicochemical profiles, including pKa, log P, etc.

Readily available, easily applicable, appropriate and validated methods to assess by single-particle optical sensing are needed to assess the stability/toxicity of an AiO PN-drug admixture. Oil immersion microscopy can be used and is a sensitive method but is hampered by the poor statistics [9,12]. As an example, a simple, sensitive, fast and cost-effective microscopic method was validated and compared with standard lipid droplet sizing methods (Coulter Counter, Photon Correlation Spectroscopy) to evaluate droplet size increase in the critical fat droplet range of 1–20 µm. In addition, even nonlipid particles (such as particulate matters or precipitates) indicating incompatibilities can be detected within the investigated size range.

Published data on stability of different medications admixed to AiO PN are frequently based on older analytical methods and techniques that no longer comply with the state-of-the-art requirements mainly based on mass spectrometry (MS) analysis [13,14,15,16,17]. Therefore, simple UV–spectrophotometric detection measures are less and less used, and the increased emergence of therapeutic drug monitoring (TDM) methods available in larger medicinal laboratories with MS detection enable faster and more specific substance characterization and quantification. Furthermore, combinations of AiO PN and medicines were not exhaustively tested nor apply to modified drug formulations, e.g., different generics available in different countries consisting of the same active pharmaceutical ingredients but of different excipients or from a different manufacturing procedure, which affect the profile of a drug, especially when it comes to more complex drugs such as biologicals or nanomedicines [18].

This study aimed to evaluate the physicochemical compatibility and stability of a lipophilic model drug, i.e., voriconazole (V)—a white crystalline powder, freely soluble in methanol and in acetonitrile, soluble in ethanol and very slightly soluble in water (97.8 mg/L), log P ≈ 1, pKa of 12.71—admixed to commercially available AiO PN admixtures under appropriate laboratory conditions [19]. Stability and compatibility data, including pH impact of V with PN are scarce, but coadministration with PN via Y-site or a separate catheter lumen is meant to be safe, although such descriptions mostly do not address the physicochemical stability, which is key for efficacy and safety and ultimately required upon modification of commercial products for individual regimen adaptation [15,20].

Moreover, the study aimed to further define and validate a simple method for pharmaceutical screening (algorithm) of drug admixtures mentioned above to timely obtain a preliminary yet reliable measure on stability of particular PN drug/additive combinations on different drug classes and supplementing an earlier evaluation published on the hydrophilic drug levetiracetam in a PN AiO admixture [21]. Such stability and compatibility questions are of highest clinical importance, often addressed on short-term notice to the pharmacists involved in (home) PN management but lacking standards for their analysis.

## 2. Materials and Methods

### 2.1. Sample Preparation

Investigations were performed at the Institute of Laboratory Medicine of the Kantonsspital Aarau between August 2017 and March 2019. Voriconazol^®^ (Pfizer PFE LOT Z464814, Switzerland GmbH containing a solubiliser, i.e., sodium–beta–cyclodextrin–sulfobutylether, stock solution 200 mg dry substance) was reconstituted according to the manufacturer’s leaflet with 19.0 mL of aqua ad inject to a final concentration of 10 mg/mL. Water, methanol (MeOH) and acetonitrile (ACN) were of LC–MS/MS grade from Sigma (Sigma Aldrich, Buchs SG, Switzerland). Three commonly used commercially available high osmolarity AiO PN admixtures containing electrolytes and zinc, mainly differing in the lipid emulsion type, were examined: SmofKabiven^®^ (SMK) 986 mL (Fresenius Kabi AG, Oberdorf, Switzerland, LOT 16LB74-2), Nutriflex^®^ Lipid Special (NLS) 625 mL (B. Braun Medical AG; Sempach, Switzerland, LOT 173238052) and Nutriflex^®^ Omega Special (OLS) 625 mL (B Braun Medical AG, Sempach, Switzerland, LOT 173238052). All products consisted of three chamber bags with separate lipid (20%), glucose (around 40%) and amino acids–electrolytes (10–15%) compartments. Table 1 shows the characteristics of the nutritional mixtures. AiO PN were prepared by breaking the seals of the bag compartment by mechanically pressing and then inverting the bag five times to homogenize.

Correlating to the common dosing of V (3–9 mg/kg body weight) for a standard patient of 80 kg, three V concentrations in the PN admixture were prepared with a manual pipette (Gilson PIPETMAN^®^, ranging from 500 to 5000 µL). Low concentration: 0.05 mg/mL (≈143.1 µM): 1.0 mL V stock solution diluted with 199 mL PN admixture; medium concentration 0.25 mg/mL (≈715.7 µM): 5.0 mL V + 195.0 mL PN; high concentration 0.5 mg/mL (≈1431.5 µM): 10.0 mL V + 190.0 mL PN; correlating to usual daily AiO volumes of 0.6–2.4 L; blanks consisted of PN admixtures without V. Test aliquots (3.0 mL) in triplicates of each concentration were transferred into transparent polystyrene tubes and sealed with polyethylene lamellar plugs. These test samples were incubated either in a refrigerator at 4 °C, at room temperature (24 °C) on the bench or at 37 °C (water bath), respectively. Test samples were not protected from light at room temperature nor in the water bath, where they were exposed to daylight or artificial light (usual laboratory conditions); test samples in the refrigerator were light-protected (closed refrigerator). Stability and compatibility tests were performed over a deliberately exaggerated period of seven days at 0, 24, 48, 72 and 168 h after admixing V in order to appropriately detect drug deterioration. To guarantee a homogenous admixture, test samples were turned upside down three times prior to analysis.

### 2.2. Visual Inspection

The test samples were inspected visually for potential discoloration, creaming, oil-in-water phase separation and precipitation at each test time point.

### 2.3. LC–MS/MS Analysis

#### 2.3.1. Pretesting

We tested a series of four dilution media for the sample preparation to match the range of the serum calibration curve. Therefore, we used pure acetonitrile, pure methanol, a mixture of 50% acetonitrile in water and a mixture of 50% methanol in water to dilute a preliminary sample of V-spiked PN, which yielded recoveries of 92%, 87%, 98% and 94%, respectively. The one with the highest recovery was selected for the following experiments.

#### 2.3.2. Testing

An LC–MS/MS instrument was used consisting of a Thermo Fisher Ultimate 3000 (U)HPLC system (Thermo Fisher, San Jose, CA, USA) coupled to a Sciex QTRAP 4500 triple quadrupole mass spectrometer (ABSciex, Darmstadt, Germany) with a commercially available AED MassTox panel kit. Chemicals for mobile phase I and II, calibration standards and quality controls, internal standard, extraction and dilution were from Chromsystems, Munich, Germany. The sample preparation had to be partially modified, as the measured V concentrations were orders of magnitude higher compared to TDM serum samples. The mass spectrometer was set to multiple reactions monitoring (MRM) mode using two mass transitions for the analyte and one for the internal standard assigned to V, a stable heavy isotope-labelled analogue of V (definitive structure not disclosed by the manufacturer). The ion source ran in positive ESI mode. As lipid-containing PN samples instead of serum samples were assessed, a predilution step with lipid extraction was developed to compensate for lipid interferences upon the analysis. A 100 µL sample aliquot was diluted 1:10 with a 50% water/acetonitrile mixture (*v/v*) for lipid extraction and was then vortexed for a few seconds and centrifuged for 5 min at 15,000× *g* in a microcentrifuge (Hettich MIKRO 20); an aliquot of the supernatant (100 µL) was diluted 1:10 again to obtain the prediluted lipid-free test sample for further sample preparation according to the manufacturer’s protocol for serum samples. Briefly, a 50 µL aliquot of the prediluted sample was transferred into a 1.5 mL reaction tube, mixed with 25 µL of the extraction buffer (composition not disclosed), agitated for ten seconds on a vortex mixer and incubated for two minutes at room temperature. Then, 250 µL of internal standards solution (composition not disclosed) were added to each tube and vortexed again for 30 s. The resulting extract was centrifuged at 15,000× *g* for five minutes, and a 100 µL aliquot of the supernatant was transferred into a glass vial and finally diluted with 600 µL of dilution buffer (composition not disclosed). The pipettes used in the range of 20–10,000 µL had to pass the lab-internal gravimetric testing (compliant with ISO 8655) within a specified accuracy of ±1.0% and a coefficient of variation of ±0.50% (*n* = 10). Matrix effects and extraction recoveries of the different spiked serum and PN samples were determined as proposed by Matuszewski et al. [22]. The injection volume was 10 µL, and the LC flow rate was 0.6 mL/min. The injection was done by a thermo-controlled autosampler at +10 °C (Dionex UltiMate 3000, Thermo Scientific, Reinach, Switzerland), and the quantification was achieved according to a weighted calibration curve (analytical measurement range: 0.02 mg/mL to 15 mg/mL, according to the manufacturer’s documentation Chromsystems (AV 92922 Antimykotika DE 06/2016 V3), normalized to the corresponding internal standard. The limit of quantification was determined to be 0.065 mg/L.

#### 2.3.3. pH Measurement

The pH measurement was conducted with a glass electrode (silver-referenced, Metrohm 744 pH Meter) for all samples. The calibration status of the instrument was checked before each measurement with a one-point quality control (pH 7.00 solution). If the control measurement deviated more than 0.04, a two-point calibration of the pH meter was performed with buffer solutions of pH 9.00 and pH 4.00 (Metrohm calibration buffer). The electrode was rinsed with distilled water (ion exchange, >15 MOhm) and dried (with a lint-free wipe) between the measurements. At the end of a sequence, the electrode was cleaned with ethanol (70% in water).

#### 2.3.4. Microscopic Evaluation

The physical stability of the *o/w* emulsion was assessed with a light transmission microscope (Olympus BX41) by measuring lipid droplets in the critical size of ≥1 µm, the lowest possible limit of detection of light microscopy. Each microscopic sample (10 µL delivered by a manual pipette on a glass slide covered with a 21 × 26 mm slip) was analysed with 1000-fold magnification and in oil immersion. For each microscopic picture, the diameter of the largest lipid droplet (LLD) and the number of lipid droplets >5 µm were assessed. The size of the lipid droplets in the visual field was determined using an ocular micrometre (0.01 mm). These stability-indicating microscopic data were measured and calculated according to a previously published method that determined specifications for a stable emulsion (Table 2) [12].

In addition to the visual counting and to automate the assessment, an electronic photo evaluation was introduced, compared and validated against visual analysis and standardized. Five photographic pictures of five individual visual fields per microscopic sample were taken in a standardized manner using a digital camera (Stingray F145C IRF IEEE1394): one picture in each corner and one in the middle of the slide (25 photos/microscopic sample). We developed a specific script for the image analysis (http://www.imageJ.net (accessed on 29 November 2017), hosted by the Laboratory for Optical and Computational Instrumentation (LOCI) of the University of Wisconsin–Madison, 2016) allowing lipid droplet counting and diameter measurements. This method was standardised and verified with a TrucountTM solution (BD MultitestTM 6-color TBNK, Becton, Dickinson and Company, BD Biosciences, San Jose, CA, USA, 23-10834-04).

### 2.4. Statistical Analysis

The statistical assessment was performed using R (version 3.5.0, The R Foundation for Statistical Computing, 2018, Vienna, Austria). Results were reported as means with standard deviations (mean ± SD) or as median and interquartile range (IQR) or as numbers and percentages (*n*, %). In addition, 95% confidence intervals, r^2^ and unpaired t-test were calculated. A *p*-value < 0.05 was considered statistically significant.

## 3. Results

### 3.1. Visual Inspection

No visual changes were observed over the seven-day testing period for the samples stored at 4 °C and at room temperature (24 °C). Neither creaming nor discolorations were detected. Samples stored at 37 °C showed yellowish discolorations after 168 h but no visible precipitates or flocculation.

### 3.2. pH Measurement

The pH of the different PN admixtures samples, including the blanks, incubated at three different temperatures showed no change over time. There was no significant difference between the examined temperatures, concentrations or manufacturers. The pH values observed were entirely between 5.48 and 5.55, with a mean ± standard deviation of 5.51 ± 0.02; SMK: 5.49 ± 0.01, 5.48–5.51; OLS: 5.52 ± 0.01, 5.51–5.55; NLS: 5.52 ± 0.01, 5.50–5.55).

### 3.3. LC–MS/MS Analysis

Extraction recoveries and potential matrix effects of PN were assessed for the three V concentrations, as the original method was specifically validated for therapeutic drug monitoring in serum samples. Since no matrix effects of PN admixture were observed, the method was deemed suitable also for drug measurements in PN AiO admixtures if the predilution steps were chosen to fit all the measurements inside of the linear measurement range.

Table 3 shows the results of the LC–MS/MS analysis. The median extraction recovery for PN samples at the lower concentration (0.05 mg/mL) was 112.5% (108.1–113.5, IQR 5.4; SMK), 108.9% (104.2–109.7, IQR 5.5; NLS) and 110.0% (106.7–110.7, IQR 4.0; OLS). For the medium concentration (0.25 mg/mL), medium extraction recovery was 102.6% (99.7–103.9, IQR 4.2; SMK), 99.0% (98.3–101.4, IQR 3.1; NLS) and 95.7% (95.1–101.5, IQR 6.3; OLS). For the high concentration (0.5 mg/mL), median extraction recovery was 98.5% (96.0–102.5, IQR 6.4; SMK), 96.0% (94.3–99.2, IQR 4.9; NLS) and 93.5% (89.4–96.5, IQR 7.1; OLS).

**Figure 1:**
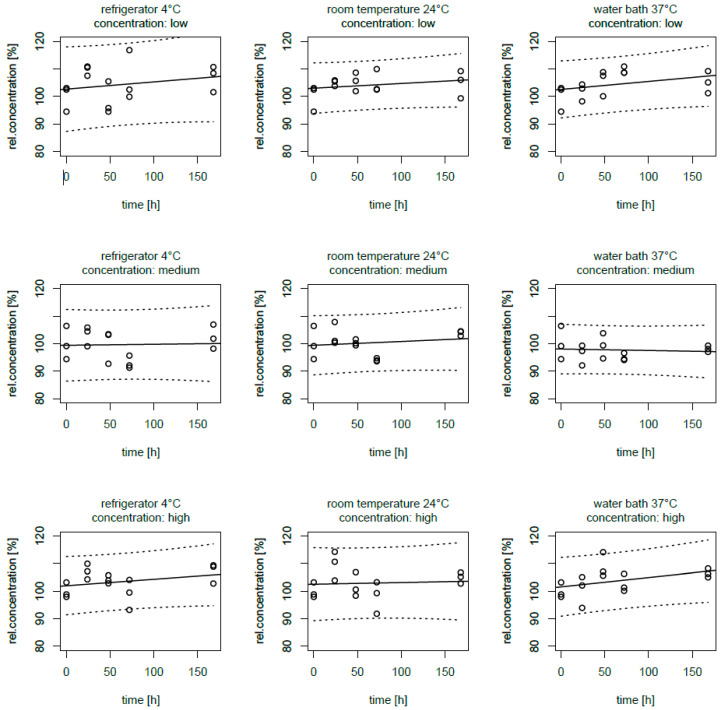
Relative voriconazole concentrations over one week (168 h) in SMK AiO PN admixtures, results of the LC–MS/MS analysis; linear fitting with prediction intervals at the specified level of 95%.

All three AiO admixtures showed a similar pattern, and there were no statistically significant differences over time, varying concentration and/or storage temperature. Figure 1 shows the relative recoveries of the SMK, stated as an example since all AiO showed the same picture.

### 3.4. Microscopic Evaluation

**Figure 2:**
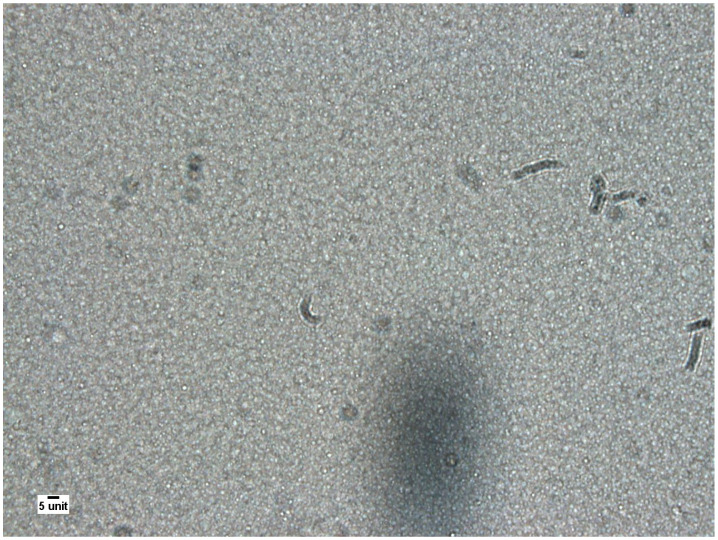
Pictures of the SMK AiO nutritional solution (1000× magnification with oil immersion), scale bar: 5 µm.

Figure 2 shows a picture taken during the microscopic evaluation. Table 4 shows the results of the visual microscopic evaluation. All samples were within the specification for the largest lipid droplets (LLD ≤ 8 µm). The largest of the LLD measured was of diameter 7.8 µm. All samples were within the specification for mean largest lipid droplet (MLLD < 4.5 µm) in the respective examined visual fields within the seven-day test period, the largest MLLD being 2.9 µm. The highest standard deviation was 1.9 µm and was thus within the specification (SDLLD ≤ 2.0 µm). All samples were within the specification for the number of droplet (LD > 5 µm ≤ 9), with the highest number being *n* = 2. There were no statistically significant differences between the blanks and the test samples and no temperature dependency.

Table 5 shows the results of the automated microscopic photo analysis evaluation. In total, 105 (89%) samples were within the specification for the largest lipid droplet (LLD ≤ 8 µm). The largest of the LLD measured was of 7.8 µm in diameter. In total, 111 (95%) samples were within the specification for mean largest lipid droplet (MLLD < 4.5 µm) in the respective examined visual fields in the seven days test period. The highest standard deviation was 3.8 µm, and 10 (8.5%) samples were outside the specification (SDLLD ≤ 2.0 µm). All samples were within the specification for the number of droplet (LD > 5 µm ≤ 9).

## 4. Discussion

We investigated the compatibility and stability of AiO PN admixtures with V in concentrations correlated to usual dosing over a deliberately exaggerated period of one week to better detect thermo-dynamic physicochemical deterioration. Only slight yellowish discoloration was seen visually after 168 h of storage at the most stressful thermal conditions (37 °C), but for both the drug containing samples and the blanks. Therefore, the coloration might most probably be due to a Maillard reaction between the amino acids and glucose (polymerisation), a commonly encountered temperature-dependent phenomenon [5]. However, we did not observe any change in pH at any time and any storage condition for all the tested AiO PN samples, possibly indicating that no measurable triglyceride hydrolysis or lipid peroxidation with creation of acidic degradation products (aldehydes oxidised to acids) occurred. The absence of trace elements may have further contributed to stable pH values, as such admixtures may catalyse deterioration of AiO admixtures by peroxidation [6]. A pH value between 5 and 9 is mandatory for lipid stability using a lecithin emulsifier for lipid emulsions and allowing a physiological lipid clearance in the blood [9,11]. A pH value below 5 may favour lipid instability [23]. Since V is a weak base, it is not excluded that it may potentially neutralise any acidic by-products produced from destabilisation of the AiO admixture. If this happened, it would not necessarily be picked up by simply measuring pH.

A commercial TDM LC–MS/MS quantification method was adapted and validated to assess physicochemical stability. LC–MS/MS methods allow simple, quick sample preparation and highly sensitive analysis with less potential for interactions and complications compared to other methods, such as HPLC. The serum sample clean-up for TDM was also appropriate for PN samples as shown in this study. All measured concentrations of V in SMK, NLS and OLS showed median recovery of 98.2% (min–max, 90–112%). The concentration variability can be explained by cumulative small errors during predilution steps. The use of internal standard compensation provides imprecision coefficients suitable for this purpose.

No significant difference was seen between the samples stored at different temperature over time mimicking even extended and normally not accepted storage times also because of potential microbial instability. The solubiliser did not affect the lipid emulsion character (blank vs. drug containing AiO PN admixtures). The tested PN products (SMK, OLS and NLS) require central venous administration (osmolarity above 1500 mOsm/L, Table 1) and represent the commonly used products in the patients with PN in Switzerland. They differ in their composition mostly in their fatty acid composition and types of lipid emulsions (long chain triglycerides, middle chain triglycerides, mono-/polyunsaturated fatty acids, fish oil). These lipid emulsions have a mean particle size corresponding to chylomicrons (approximately 0.25–0.5 µm). Larger lipid droplets (fat globules > 5 µm) potentially blocking small blood capillaries were almost absent [9,24].

The 1000-fold magnification in the microscopic analysis allows the detection of critical particles between 1 and 20 µm in diameter as well as other nonlipid particles e.g., precipitates. The validated and sensitive, conventional microscopic method from Schmutz fits the purpose and requires only simple equipment [12]. In contrast to other particle counting and size assessment methods, like laser-based counting or photo correlation methods, used especially during industrial manufacturing assessment, it allows the analysis of undiluted PN samples and detection of the clinically critical oil droplet sizes. This eliminates an important dilution bias when assessing critical droplet sizes (≥1–2 um), and is therefore, especially suitable or necessary for drug incompatibility testing of AiO PN admixtures [12]. When comparing this microscopic single particle analysis with the USP method 729 requesting a PFAT5 < 0.05%, the given limits and results of MLLD < 4.5 ± 2.0 µm, LLD, LD > 5 µm ≤ 9. We conservatively estimated PFAT5 in our samples (all assumptions were made such that they would result in the largest possible PFAT5) and found that PFAT5 was well below 0.05% in all samples.

No significant change in the distribution range of the lipid droplets over the test period was detected with the exception of a slight, but not significant increase in MLLD at 37 °C (stress condition). Our results show no dependence of the fatty acid composition for the lipid emulsion stability. All parameters were within the specifications. However, potential slip of the droplets during handling may affect their actual size and droplets may for example overlap or agglomerate. Nevertheless, we did not find indication for this phenomenon in our data.

As the visual microscopic assessment represents a conventional method with potential interindividual deviations by the operator, we introduced and validated a computer software script for automated electronic detection of the globules from digital photos. The data for both methods were comparable (Table 4 and Table 5) after removing the pictures showing air bubbles, since this could not be solved with the software script. The automated computer method is convenient and time-saving for the practical application and allows an increased throughput of analyses of microscopic pictures to further improve the statistics. Additionally, the pictures taken can be stored and serve as documents or references for possible later requests. The developed method must be further developed and tested for reliability since accurate droplet segmentation is challenging.

The microscopic test setting would also implicate stability for Y-site administration of a drug and PN. The analysis could probably be reduced to the lipid emulsion characterization and a shorter observation period.

As this study with V and AiO PN used glass and polystyrene containers instead of original packaging, we have no data on potential drug adsorption to the PN bag or line materials. This should be minimal as long as V is solubilized in the admixtures, as it was not extracted by the lipid clean-up procedure, indicating the presence of solubilized V in the water phase. This must be considered in further investigations. For a more complete investigation of the compatibility of such PN-drug admixtures and the outcome of both nutrition and pharmacological treatment on the patient, a clinical study would be the gold standard to show also the clinical therapeutic effect. Nevertheless, showing the pharmaceutical quality of the individual formulation is a mainstay and prerequisite to guarantee optimal treatment for patients.

In this study, there were no V-specific effects on the AiO PN admixture measurable in vitro. Compatibility and stability of the lipophilic, solubilized V of the branded drug product in a dose range of 0.05–0.5 mg/mL (143–1431 µM) in the commercial AiO admixtures investigated can be concluded for one week as no concentration change occurred.

The investigation of this AiO PN drug admixture was done over 168 h. As V was the active pharmaceutical ingredient combined with other ingredients from the specific branded drug formulation, results from another V formulation (e.g., generics) with changed composition may potentially differ; this has to be considered upon extrapolation of the data. Compatibility applications cannot be transferred to various commercial preparations. An additional limitation was the used PN regimens without micronutrients (vitamins or trace elements) added except zinc, which are required in a complete PN and commonly used in clinical practice. Trace elements and vitamins can, however, significantly affect the compatibility and stability of PN solution itself and of the added drug. It would therefore be suited to do additional tests with these micronutrients admixtures to further contribute to a convenient and simplified and more complete ready-to-use administration of the specific AiO drug regimen.

Related to the second aim of the study, the results indicate and further support the useful approach to check stability and compatibility of drugs by using a TDM-adapted method such as LC–MS/MS together with a relatively simple microscopic analysis to check the AiO PN emulsion characteristics compatible with the USP requirements on PFAT5 < 0.05%. Such validated and available setting allows a timely response, almost within 24–48 h, for a specific PN drug compatibility assessment with a proper data-supported documentation of the pharmaceutical quality of an individual, patient-specific PN drug admixture. Such a PN drug evaluation setup would be preferably available in tertiary care hospitals where expertise and collaboration between the clinical laboratory (established and validated TDM methods) and the pharmacy with the necessary specific PN analytics exist or can be established and where daily practical exposure for larger cohorts of PN patients are monitored. The investigations done so far have added to define robust, easy-to-do and validated laboratory methods to evaluate drug PN interactions timely [21]. They also contribute to the understanding how physicochemical characteristics of a drug and the formulation—e.g., with a solubilized lipophilic drug—correlate with potential AiO PN emulsions’ stability issues. This supports an approach to classify the drugs and their formulations physicochemically to create a useful checklist for pharmaceutical drug PN stability requests addressed to the hospital pharmacy. A checklist based on such findings allows an initial assessment and advice before starting laboratory analysis. Such evaluation methods would also assist to define standards for drugs to be admixed in hospitals where defined ready-to-use AiO PN admixtures are frequently prescribed and the number of concomitant drug therapies for potential coadministration into PN is limited. Ultimately, such defined procedures add to render a PN and drug regimen more convenient and less risky by easier handling in selected cases. They also address responsibility for the involved health care professionals, especially the pharmacist, by documenting the stability and safety of the admixtures concerned independently from whether commercial or tailor-made PN admixtures are used. Such procedures and documentation would be compliant with existing PN guidelines, e.g., in-the-home care and outpatient nutritional support [25].

## 5. Conclusions

We investigated the compatibility and stability of a commercially branded V, a poorly water-soluble, lipophilic and weakly basic drug solubilized with sodium–beta–cyclodextrin–sulfobutylether added to AiO PN admixtures (SMK/NLS/OLS), in three concentrations (0.05/0.25/0.5 mg/mL) at different storage temperatures (4/24/37 °C). The data showed compatibility and stability over one week. Using computer software script to assess lipid emulsion characteristics from microscopic photos allowed switching from a labour-bound and time-consuming visual method to an automated one as shown by the corresponding evaluation. The test method proposed is useful in tertiary care hospitals where a central medicinal laboratory with TDM methods is established together with pharmaceutical drug product analysis expertise to scientifically document the compatibility and stability of drug PN admixtures necessary for convenient patient care. This furthermore eliminates important medication errors.

## Figures and Tables

**Table 1:** Composition of the industrially compounded parenteral nutritional solutions.

AiO admixture	SmofKabiven (SMK)	Nutriflex Lipid Special (NLS)	Nutriflex Omega Special (OLS)
Manufacturer	Fresenius Kabi AG	B. Braun Medical AG	B. Braun Medical AG
**Volume (14)**	986	625	625
**Glucose [g]**	125	90	90
**Amino acid [g]**	50	35	35.9
**Fat [g]**	38	25	25
**Total energy [kcal]**	1100	738	740
**Nonprotein Energy [kcal]**	900	600	600
**Nitrogen [g]**	8	5	5
**Osmolarity [mOsmol/L]**	1500	1545	1545
**pH**	5.6	5–6	5–6
**Ratio kcal/g N**	113	120	120
**Fat [g]**			
Soybean oil (LCT)	11.5	12.5	10
Olive oil (LCT)	9.5	N/A	N/A
Fish oil (LCT)	5.7	N/A	N/A
Medium-Chain Triglycerides	11.5	12.5	12.5
Egg lecithin	N/A	1.5	N/A
Ω-3 fatty acids	2.5	N/A	2.5 g
Monounsaturated fatty acids	N/A	13	11.4
Polyunsaturated fatty acids	N/A	30.7	34.0
Ratio Ω-3:Ω-6	1:2.5	1:7	1:2.7
Essential fatty acids	N/A	30.7	31.7
**Electrolyte [mmol]**			
Sodium	41	33.5	33.5
Potassium	30	23.5	23.5
Magnesium	5.1	2.65	2.65
Calcium	2.5	2.65	2.65
Phosphate	12.2	10	10
Chloride	35.5	30	30
Acetate	105.5	30	30
Zinc	0.04	0.02	0.02
Sulphate	5.1	N/A	N/A

Abbreviations: AiO admixture, all-in-one admixture; N/A, not applicable; Ω-3, Omega three; Ω-6, Omega six.

**Table 2:** Specifications of microscopic intravenous fat emulsion stability assessment (12).

Microscopic Parameter	Abbreviation	Unit	Specification
Largest lipid droplet in 15 fields	LLD1-15	[µm]	≤8
Mean largest lipid droplet	MLLDmax	[µm]	<4.5
Standard deviation of the largest lipid droplets	SDLLD	[µm]	≤2
Number of lipid droplets >5 um	LD > 5 µm	[n]	≤9

**Table 3:** Voriconazole concentrations over one week (168 h) in different AiO PN admixtures, results of the LC–MS/MS analysis.

AiO Admix			SMK					NLS					OLS				
Hours			0	24	48	72	168	0	24	48	72	168	0	24	48	72	168
**KS**	**L**	mv	53.6	58.7	52.8	57.0	57.3	52.8	56.1	51.0	54.5	51.4	53.4	56.7	55.6	55.2	53.4
		rsd	3.92	1.37	4.98	7.02	3.64	2.28	3.50	2.03	3.22	2.53	5.18	3.46	3.32	2.07	6.81
	**M**	mv	256	264	256	238	262	247	246	244	246	231	238	253	257	239	221
		rsd	4.97	2.85	5.03	2.06	3.52	1.12	0.52	4.58	2.74	1.65	3.97	2.29	4.24	2.12	6.14
	**H**	mv	480	514	500	475	514	480	469	473	474	430	447	433	438	472	465
		rsd	2.30	2.14	1.15	4.53	2.79	2.71	3.97	3.29	2.04	3.00	7.59	11.2	10.5	4.59	4.72
**RT**	**L**	mv	53.6	56.3	56.5	56.3	56.2	52.8	56.0	54.8	53.5	50.2	53.4	54.8	56.9	53.4	55.4
		rsd	3.92	0.85	2.61	3.31	3.93	2.28	4.60	3.16	3.67	3.34	5.18	0.39	2.12	3.99	4.50
	**M**	mv	256	264	257	241	266	247	251	255	254	231	238	248	232	241	254
		rsd	4.97	3.32	0.92	0.51	0.73	1.12	0.85	3.87	1.32	3.74	3.97	3.55	3.53	1.53	2.38
	**H**	mv	480	526	489	471	504	480	496	496	487	468	447	484	465	484	469
		rsd	2.30	3.92	3.54	4.85	1.56	2.71	0.62	3.46	3.91	1.42	7.59	0.77	4.14	5.77	12.2
**WB**	**L**	mv	53.6	54.5	56.5	58.6	56.3	52.8	54.4	54.9	56.6	49.1	53.4	55.3	53.9	55.3	52.3
		rsd	3.92	2.58	3.66	0.97	3.12	2.28	1.33	1.36	4.49	3.12	5.18	4.61	0.56	2.54	3.31
	**M**	mv	256	246	254	243	251	247	252	257	257	219	238	236	255	264	237
		rsd	4.97	3.17	3.78	1.16	0.96	1.12	2.16	0.14	0.63	4.87	3.97	2.28	2.78	10.2	3.57
	**H**	mv	480	482	523	492	511	480	497	501	513	416	447	481	486	484	467
		rsd	2.30	4.70	3.41	2.59	1.25	2.71	2.77	1.69	3.01	3.40	7.59	6.52	0.68	0.16	2.11

SMK, Smofkabiven; NLS, Nutriflex lipid special; OLS, Nutriflex omega special; KS, fridge; RT, room temperature; WB, water bath; L, low V concentration (0.05 mg/mL); M, medium V concentration (0.25 mg/mL); H, high V concentration (0.5 mg/mL); mv, mean value; rsd, relative standard deviation (%).

**Table 4:** Results of the visual microscopic analysis (size in µm and counts).

			SMK					NLS					OLS				
		Hours	0	24	48	72	168	0	24	48	72	168	0	24	48	72	168
**MLLD**																	
**KS**	**L**	mv	-	0.0	3.1	2.9	0.0	-	1.4	1.3	1.4	1.4	-	1.3	1.9	1.6	1.4
	**M**	mv	-	0.0	2.9	2.6	2.7	-	1.6	1.5	1.3	1.3	-	1.5	2.1	1.4	1.3
	**H**	mv	-	0.0	2.7	2.6	2.7	-	1.8	1.6	1.6	1.7	-	1.3	1.3	1.3	1.7
**RT**	**L**	mv	2.7	0.0	2.6	2.6	2.6	1.7	1.7	1.3	1.5	1.6	1.5	1.3	1.4	1.3	1.6
	**M**	mv	2.6	0.0	2.6	0.0	2.6	2.0	1.5	1.6	1.6	1.6	1.5	1.6	1.8	1.3	1.5
	**H**	mv	2.6	0.0	2.6	2.6	0.0	1.9	1.3	1.6	1.3	1.7	1.3	1.5	1.8	1.3	1.7
**WB**	**L**	mv	-	2.6	2.6	3.0	2.6	-	1.9	1.6	1.7	1.7	-	1.3	1.4	1.8	2.6
	**M**	mv	-	2.6	2.6	2.8	2.6	-	1.8	1.6	2.1	1.7	-	1.3	2.3	1.4	2.6
	**H**	mv	-	3.0	2.6	2.9	0.0	-	1.4	1.3	1.8	3.0	-	1.8	1.4	1.3	2.4
**LLD**																	
**KS**	**L**	mv	-	0.0	5.2	3.9	0.0	-	2.6	1.3	2.6	2.6	-	1.3	2.6	2.6	2.6
	**M**	mv	-	0.0	3.9	2.6	3.9	-	2.6	2.6	1.3	1.3	-	2.6	2.6	2.6	1.3
	**H**	mv	-	0.0	3.9	2.6	3.9	-	5.2	5.2	5.2	7.8	-	1.3	1.3	1.3	3.9
**RT**	**L**	mv	3.9	0.0	2.6	2.6	2.6	2.6	5.2	1.3	2.6	3.9	2.6	1.3	2.6	1.3	5.2
	**M**	mv	2.6	0.0	2.6	0.0	2.6	2.6	2.6	2.6	5.2	5.2	2.6	2.6	3.9	1.3	2.6
	**H**	mv	2.6	0.0	2.6	2.6	0.0	5.2	1.3	2.6	1.3	5.2	1.3	2.6	3.9	1.3	5.2
**WB**	**L**	mv	-	2.6	2.6	3.9	2.6	-	5.2	2.6	6.5	3.9	-	1.3	2.6	3.9	3.9
	**M**	mv	-	2.6	2.6	3.9	2.6	-	7.8	2.6	7.8	5.2	-	1.3	2.6	2.6	5.2
	**H**	mv	-	3.9	2.6	3.9	0.0	-	2.6	1.3	5.2	6.5	-	2.6	2.6	1.3	5.2
**SD_LLD_**																	
**KS**	**L**	std	-	0.0	0.8	0.0	0.0	-	0.3	0.0	0.3	0.3	-	0.0	0.6	0.6	0.3
	**M**	std	-	0.0	0.6	0.0	0.4	-	0.6	0.4	0.0	0.0	-	0.5	0.6	0.4	0.0
	**H**	std	-	0.0	0.4	0.5	0.3	-	1.0	1.0	1.0	1.6	-	0.0	0.0	0.0	0.7
**RT**	**L**	std	0.4	0.0	0.0	0.0	0.0	0.6	1.1	0.0	0.4	0.7	0.4	0.0	0.3	0.0	1.0
	**M**	std	0.0	0.0	0.0	0.0	0.0	0.6	0.5	0.5	1.0	1.0	0.4	0.6	0.8	0.0	0.4
	**H**	std	0.0	0.0	0.0	0.0	0.0	1.0	0.0	0.5	0.0	1.0	0.0	0.5	0.8	0.0	1.0
**WB**	**L**	std	-	0.0	0.0	0.6	0.0	-	1.1	0.6	1.3	0.7	-	0.0	0.3	0.9	0.8
	**M**	std	-	0.0	0.0	0.5	0.0	-	1.6	0.6	1.6	1.1	-	0.0	0.5	0.4	0.8
	**H**	std	-	0.6	0.0	0.5	0.0	-	0.3	0.0	1.1	2.0	-	0.6	0.3	0.0	1.3
**LD > 5 µm**																	
**KS**	**L**	n	-	0	2	0	0	-	0	2	0	0	-	0	0	0	0
	**M**	n	-	0	0	0	0	-	0	0	0	0	-	0	0	0	0
	**H**	n	-	0	0	0	0	-	1	1	1	1	-	0	0	0	0
**RT**	**L**	n	0	0	0	0	0	1	1	0	0	0	0	0	0	0	1
	**M**	n	0	0	0	0	0	0	0	0	1	1	0	0	0	0	0
	**H**	n	0	0	0	0	0	1	0	0	0	1	0	0	0	0	1
**WB**	**L**	n	-	0	0	0	0	-	1	0	1	0	-	0	0	0	0
	**M**	n	-	0	0	0	0	-	1	0	1	1	-	0	0	0	1
	**H**	n	-	0	0	0	0	-	0	0	1	2	-	0	0	0	2

H, high V concentration (0.5 mg/mL); KS, fridge; L, low V concentration (0.05 mg/mL); LD, lipid droplets; LLD, largest lipid droplet; M, medium V concentration (0.25 mg/mL); MLLD, mean largest lipid droplets; mv, mean value; n: number; NLS, Nutriflex lipid special; OLS, Nutriflex omega special; RT, room temperature; SD_LLD_, standard deviation of the largest lipid droplets; SMK, Smofkabiven; std, standard deviation; WB, water bath.

**Table 5:** Results of the automated microscopic photo analysis (size in µm and counts).

			SMK					NLS					OLS				
		Hours	0	24	48	72	168	0	24	48	72	168	0	24	48	72	168
**MLLD**																	
**KS**	**L**	mv	-	2.7	2.9	2.9	4.1	-	3.2	2.7	2.7	2.9	-	2.3	4.9	0.0	2.2
	**M**	mv	-	2.1	3.0	3.0	2.9	-	2.1	2.4	2.4	3.2	-	3.2	3.3	3.3	3.9
	**H**	mv	-	2.4	3.0	3.0	2.6	-	3.9	2.6	2.6	0.0	-	2.3	3.6	3.6	3.6
**RT**	**L**	mv	2.6	2.1	3.3	3.3	3.4	2.7	2.4	3.2	3.2	3.3	3.0	2.7	2.1	2.1	3.1
	**M**	mv	2.4	2.1	2.2	2.2	2.7	2.4	2.5	3.2	3.2	4.5	3.4	3.4	2.1	2.1	2.6
	**H**	mv	2.6	4.2	4.9	0.0	2.2	2.6	2.4	5.3	0.0	2.5	3.5	2.6	2.5	2.5	2.4
**WB**	**L**	mv	-	2.1	2.5	2.5	14	-	2.7	2.4	2.4	3.2	-	3.7	3.4	3.4	2.8
	**M**	mv	-	2.8	2.7	2.7	3.5	-	3.9	3.4	3.4	2.7	-	2.5	3.5	3.5	3.1
	**H**	mv	-	2.0	4.1	4.1	3.2	-	2.8	2.9	3.0	4.5	-	2.7	3.6	3.6	6.3
**LLD**																	
**KS**	**L**	mv	-	2.7	4.6	4.6	0.0	-	4.8	3.0	3.0	3.9	-	2.6	15	0.0	2.4
	**M**	mv	-	2.2	3.7	3.7	6.9	-	2.1	2.6	2.6	7.5	-	5.6	5.8	5.8	9.7
	**H**	mv	-	3.2	4.1	4.1	4.0	-	14	3.7	3.7	0.0	-	2.7	4.8	4.8	12
**RT**	**L**	mv	3.3	2.3	3.9	3.9	7.7	3.0	3.0	5.5	5.5	3.8	4.1	4.5	2.1	2.1	5.4
	**M**	mv	2.7	2.2	2.2	2.2	3.3	2.6	2.7	3.8	3.8	4.5	3.5	7.7	2.3	2.3	3.7
	**H**	mv	3.7	6.0	9.0	0.0	2.6	3.7	3.0	10	0.0	2.8	0.0	3.2	3.0	3.0	2.8
**WB**	**L**	mv	-	2.2	3.4	3.4	26	-	5.7	3.0	3.0	7.8	-	4.7	6.1	6.1	5.8
	**M**	mv	-	3.2	3.5	3.5	9.3	-	7.4	5.1	5.1	6.5	-	2.8	5.3	5.3	5.8
	**H**	mv	-	2.1	14	0.0	9.7	-	3.5	3.9	3.9	9.3	-	3.5	4.0	4.0	16
**SD_LLD_**																	
**KS**	**L**	std	-	0.0	0.9	0.9	0.0	-	1.3	0.3	0.3	0.7	-	0.4	4.6	0.0	0.2
	**M**	std	-	0.1	0.9	0.9	1.7	-	0.0	0.3	0.3	1.7	-	0.9	1.2	1.2	2.6
	**H**	std	-	0.4	0.6	0.6	0.6	-	3.4	1.0	1.0	0.0	-	0.3	0.8	0.8	2.9
**RT**	**L**	std	0.4	0.2	0.6	0.6	2.4	0.3	0.3	1.2	1.2	0.4	0.8	1.0	0.0	0.0	1.6
	**M**	std	0.2	0.1	0.0	0.0	0.6	0.3	0.2	0.8	0.8	0.0	0.1	2.2	0.1	0.1	0.5
	**H**	std	0.9	2.6	3.4	0.0	0.4	1.0	0.4	4.1	0.0	0.4	0.0	0.5	0.4	0.4	0.4
**WB**	**L**	std	-	0.1	0.6	0.6	16	-	1.0	0.3	0.3	1.5	-	1.0	1.5	1.5	1.1
	**M**	std	-	0.6	0.7	0.7	1.8	-	3.1	1.6	1.6	1.0	-	0.4	1.2	1.2	1.0
	**H**	std	-	0.1	3.9	0.0	1.6	-	0.4	0.7	0.7	2.6	-	0.4	0.3	0.3	4.9
**LD > 5 µm**																	
**KS**	**L**	n	-	2	2	2	2	-	3	3	3	3	-	5	1	1	5
	**M**	n	-	1	1	1	1	-	1	1	1	1	-	1	2	2	6
	**H**	n	-	1	1	1	1	-	3	3	3	0	-	1	1	1	1
**RT**	**L**	n	2	1	1	1	1	3	3	1	1	3	1	1	1	1	1
	**M**	n	1	1	1	1	1	1	1	1	1	1	2	1	1	1	2
	**H**	n	1	1	2	2	1	3	3	2	2	3	1	1	1	1	1
**WB**	**L**	n	-	1	1	1	1	-	1	1	1	1	-	2	1	1	2
	**M**	n	-	3	3	3	3	-	1	1	1	1	-	2	1	1	2
	**H**	n	-	8	2	2	8	-	3	3	3	3	-	5	5	5	5

H, high V concentration (0.5 mg/mL); KS, fridge; L, low V concentration (0.05 mg/mL); LD, lipid droplets; LLD, largest lipid droplet; M, medium V concentration (0.25 mg/mL); MLLD, mean largest lipid droplets; mv, mean value; n, number; NLS, Nutriflex lipid special; OLS, Nutriflex omega special; RT, room temperature; SD_LLD_, standard deviation of the largest lipid droplets; SMK, Smofkabiven; std, standard deviation; WB, water bath.

## Data Availability

Data is contained within the article.
